# Localised JAK/STAT Pathway Activation Is Required for *Drosophila* Wing Hinge Development

**DOI:** 10.1371/journal.pone.0065076

**Published:** 2013-05-31

**Authors:** Kirsty Johnstone, Richard E. Wells, David Strutt, Martin P. Zeidler

**Affiliations:** MRC Centre for Developmental and Biomedical Genetics and the Department of Biomedical Science, The University of Sheffield, Sheffield, United Kingdom; University College London, United Kingdom

## Abstract

Extensive morphogenetic remodelling takes place during metamorphosis from a larval to an adult insect body plan. These changes are particularly intricate in the generation of the dipteran wing hinge, a complex structure that is derived from an apparently simple region of the wing imaginal disc. Using the characterisation of original *outstretched* alleles of the *unpaired* locus as a starting point, we demonstrate the role of JAK/STAT pathway signalling in the process of wing hinge development. We show that differences in JAK/STAT signalling within the proximal most of three lateral folds present in the wing imaginal disc is required for fold morphology and the subsequent differentiation of the first and second auxiliary sclerites as well as the posterior notal wing process. Changes in these domains are consistent with the established fate map of the wing disc. We show that *outstretched* wing posture phenotypes arise from the loss of a region of Unpaired expression in the proximal wing fold and demonstrate that this results in a decrease in JAK/STAT pathway activity. Finally we show that reduction of JAK/STAT pathway activity within the proximal wing fold is sufficient to phenocopy the *outstretched* phenotype. Taken together, we suggest that localised Unpaired expression and hence JAK/STAT pathway activity, is required for the morphogenesis of the adult wing hinge, providing new insights into the link between signal transduction pathways, patterning and development.

## Introduction

**Figure 1:**
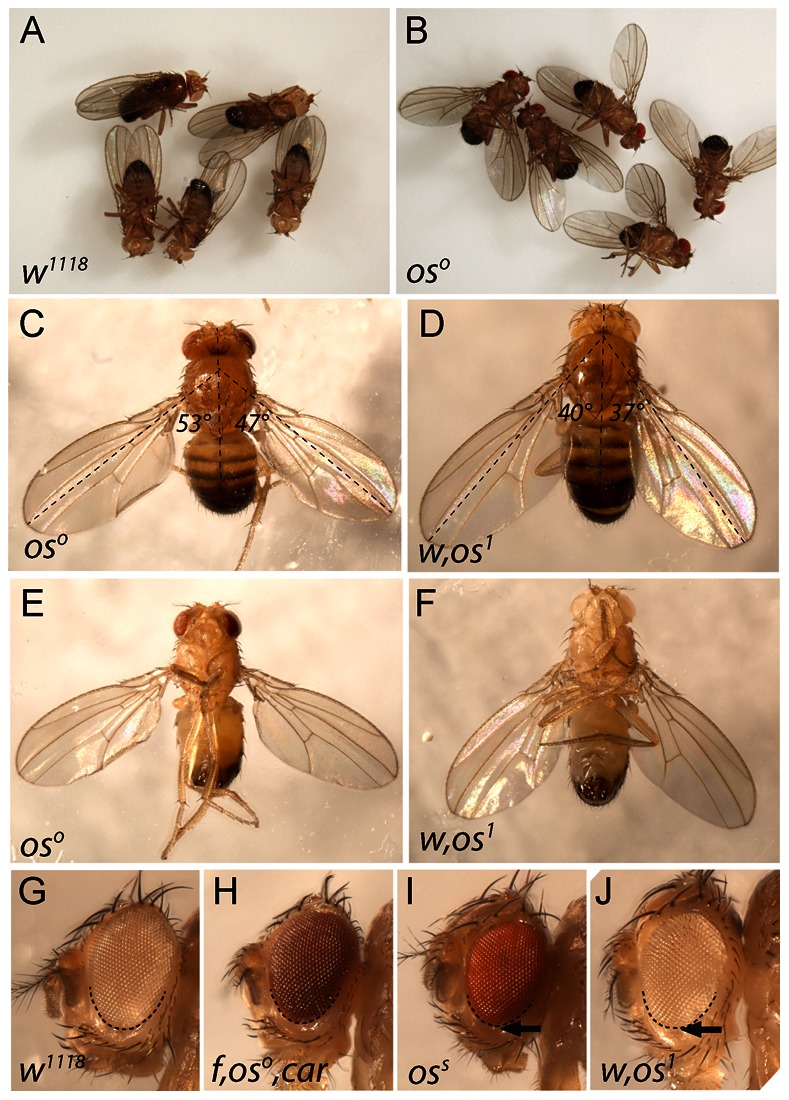
The *outstretched* wing phenotype. (**A**) Wild type (*w^1118^*) adult male *Drosophila melanogaster* showing typical resting wing posture. (**B**) Adult males hemizygous for the *os^o^* allele showing their typical resting wing posture. (**C–F**) Adult males of the indicated hemizygous genotypes showing resting wing postures typically associated with the outstretched phenotype. Dorsal (C,D) and ventral (E–F) views are shown. The angle of the wing relative to the body axis (dotted lines) is indicated. (**G–J**) Eyes of the indicated genotypes. Extent of ventral eye field is outlined by dashed line. Minor ventral eye size reduction (arrows) is visible in *os^s^* and *os^1^* alleles.

First characterised over 80 years ago, the *outstretched (os)* phenotype represents the first JAK/STAT pathway-related defect to be genetically described [1]. Originally named on the basis of the distinctive outstretched wing posture of adults (Figure 1A–F), other alleles with small eyes (*os^s^*; Figure 1I) or both small eyes and outstretched wings (Figure 1D,F & J) were subsequently identified [2]. Some years later, mutations in the *unpaired (upd)* locus were independently identified on the basis of their atypical embryonic pair-rule segmentation defects [3] and complementation experiments demonstrated that strong *upd* alleles fail to complement the original viable *os* alleles, suggesting that both represent mutations in the same locus [4], [5].

Using embryonic cuticle clonal analysis, genetic characterisation of strong *upd* alleles demonstrated that the associated gene encodes a protein able to act non-autonomously during development [6]. Consistent with this, molecular cloning showed that *upd* encodes a secreted and glycosylated protein able to act as a JAK/STAT pathway ligand [7], [8]. More recently, two additional Upd-like ligands termed Upd2 and Upd3 have also been identified. All three ligands are able to stimulate pathway signalling both *in vivo* and in cell based assays [9] with *upd* and *upd2* being expressed in similar patterns and functioning semi-redundantly during development [10].

As shown both *in vivo* and in cell culture-based systems, Upd ligands bind to the Domeless cytokine receptor [11], [12], causing the activation of the JAK tyrosine kinase homologue Hopscotch [4], [13] which leads to phosphorylation of the *Drosophila* STAT92E transcription factor [14], [15]. Once activated, STAT92E translocates into the nucleus where it binds a palindromic DNA sequence present in the promoter of target genes and activates transcription. Functionally, the *Drosophila* JAK/STAT pathway is involved in multiple developmental processes including haematopoiesis, immune responses, cell proliferation and stem cell maintenance (reviewed in [16]–[18]). In addition, studies into the role of JAK/STAT signalling in the developing *Drosophila* eye have shown that reduced pathway activity results in cellular under proliferation [19] to generate a phenotype similar to the viable *os^s^* and *os^1^* small eye phenotype (Figure 1G–J).

Here we present an analysis of the previously uncharacterised role for *upd* expression in the developing wing hinge as demonstrated by *outstretched* alleles. We show that the development of adult structures on the dorsal hinge requires the most proximal of three folds present within the third instar wing imaginal disc. Furthermore, formation of this fold requires localised *upd* expression, which is lacking in *os* alleles. Using *in vivo* RNAi approaches, we also show that reducing JAK/STAT pathway activity can phenocopy the held out wing defect associated with these previously uncharacterised classical *os* alleles.

## Results and Discussion

### The Outstretched Phenotype

Wild type adult *Drosophila melanogaster* generally maintain a characteristic resting wing posture where wings are held almost parallel to the main body axis (Figure 1A). By contrast, males hemizygous for the *outstretched (os)* alleles *os^o^* and *os^1^* hold their wings out at 30–80° from the principal body axis (Figure 1B–F) but have apparently normal wing and body morphology (Figure 1C–F). In addition males hemizygous for *os^1^* and *os^s^* also have mildly reduced ventral eye size (arrows in Figure 1I,J) compared to wild type and *os^o^* alleles (Figure 1G,H). However, the wing posture of *os^s^* flies is normal.

**Figure 2:**
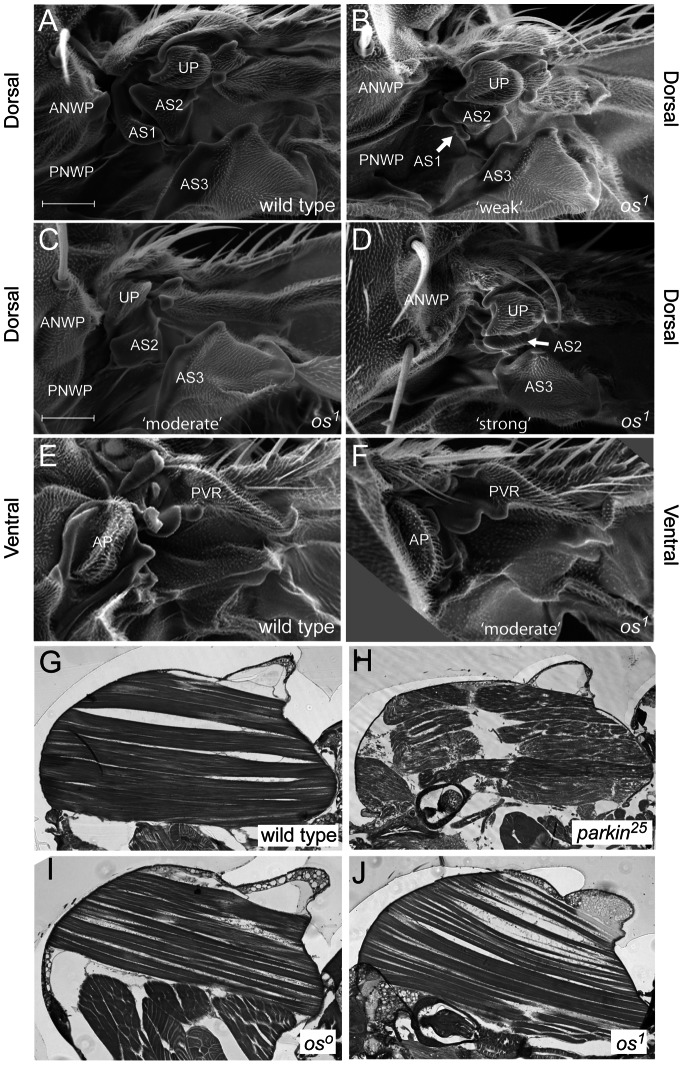
Structure of the *os^1^* hinge and adult musculature. Scanning electron micrographs of the adult dorsal (**A–D**) and ventral (**E–F**) wing hinge regions of wild type (A, E) and *os^1^* (B–D and F) individuals. The relative expressivity of the *os^1^* allele phenotypes is indicated by ‘weak’ ‘moderate’ and ‘strong’ labels. Note how increasing phenotypic strength is associated with the progressive loss of the AS1, AS2 and PNWP. Wing hinge structures are named in [25]. Unnamed plate (UP), first, second and third auxiliary sclerites (AS1, AS2 and AS3), anterior notal wing process (ANWP) and posterior notal wing process (PNWP) are present in the dorsal hinge. In the ventral hinge, auxillary pouch (AP) and proximal ventral radius (PVR) are shown. (**G–J**) Longitudinal sections through adult males of the indicated genotypes show the structure of the primary flight muscles.

In order to better understand the basis of the *os* wing posture phenotype we examined the hinge area of adult flies. By comparison to wild type males (Figure 2A), individuals hemizygous for both *os^o^* and *os^1^* alleles show distinct dorsal hinge defects. Flies carrying the *os^1^* allele have variable expressivity: those with a relatively ‘weak’ held out phenotype have a reduced/rudimentary first auxiliary sclerite (AS1) (arrow in Figure 2B). Moderately affected individuals lack the AS1 completely (Figure 2C) while individuals with the strongest expressivity lack AS1 and, in addition, the AS2 and AS3 are reduced as is the posterior notal wing process (PNWP) (Figure 2D). However, adjacent structures such as the unnamed plate (UP) and the anterior notal wing process (ANWP) appear normal in all individuals. By contrast to the differences apparent on the dorsal surface of the hinge, ventral regions of both wild type and moderate *os^1^* alleles appear to be similar (Figure 2E–F).

An alternative cause of wing posture phenotypes in *Drosophila* are defects in the primary flight musculature, one example of which are homozygous *parkin* mutants (compare Figure 2G and 2H) [20]. However, longitudinal sections through both *os^o^* and *os^1^* males (Figure 2I–J) show muscle structures similar to wild type (Figure 2G) suggesting that the wing posture defect in these alleles is not a consequence of a gross defect in flight muscle development.

Taken together, these results suggest that defects in the wing hinge, and the auxiliary sclerites, and AS1 in particular, are the basis of the wing posture defects observed in *os^1^* and *os^o^* mutants.

### Outstretched Mutant Wing Discs

The adult wing, hinge region and dorsal thoracic body wall are derived from the wing imaginal disc, a sheet of columnar epithelial cells set aside during embryogenesis [21]. During larval life, cells within each wing imaginal disc proliferate and grow and by late third instar the disc contains three prominent transverse folds spanning the presumptive dorsal hinge region. During pupariation the wing imaginal discs undergo a series of complex morphological rearrangements termed eversion that ultimately give rise to the adult structures [22].

**Figure 3:**
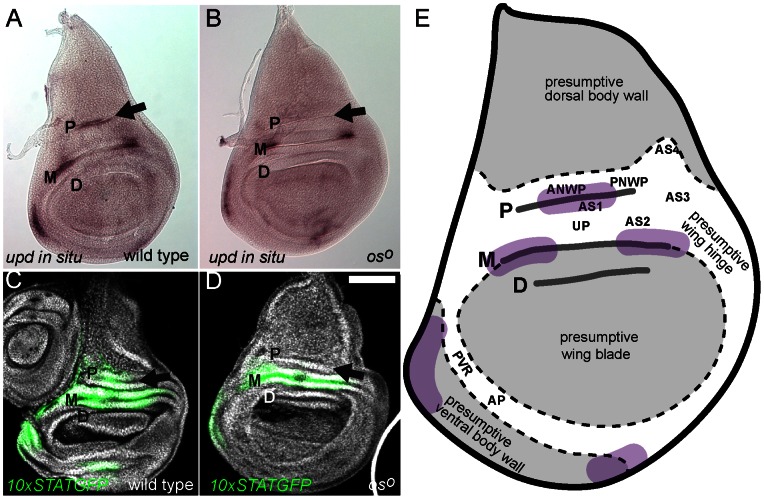
Expression of *upd* and activation of JAK/STAT pathway signalling in the wing disc. Proximal (P), medial (M) and distal (D) folds are labelled. Scale bar is 100 µm, (**A** & **B**) Third instar wing imaginal discs showing the expression of *upd* mRNA in wild type (A) and *os^o^* (B) individuals. Expression of *upd* within the P fold (arrow) is lost in the *os^o^* mutant. (**C** & **D**) Third instar wing imaginal discs showing DNA (white) and the *10xSTATGFP* reporter (green) in wild type (C) and *os^o^* (D) individuals. Reporter activity within the P fold (arrow) is greatly reduced in *os^o^* mutants suggesting that JAK/STAT pathway ligands are not expressed in this area. (**E**) Schematic representation of the third instar wing imaginal disc fate map showing the regions labelled in Figure 2, the P, M and D lateral folds and the approximate regions of wild type *upd* expression (purple). Image based on fate map originally published in [25].

Given the correlation between *os^1^* wing posture phenotypes and the loss of dorsal wing hinge structures shown above, we examined the expression of *upd* mRNA within the third instar wing imaginal disc. In wild type individuals, *upd* is expressed within three regions of the presumptive dorsal wing hinge as well as in the anterior body wall and, at lower levels, in the ventral body wall (purple regions in Figure 3A and 3E). By contrast, in *os^o^* wing discs the most proximal dorsal region of *upd* expression is no longer detected (arrow in Figure 3B), although other expression domains appear normal.

In vitro reporters containing multiple STAT92E binding sites upstream of a GFP coding region allow the spatial and temporal pattern of JAK/STAT pathway activity elicited by Upd-like ligands to be visualised during development [9], [23]. We used one such *10xSTATGFP* reporter to identify regions of pathway activation within the third instar wing imaginal disc. Consistent with *upd* mRNA expression, *10xSTATGFP* reporter activity in wild type wing discs is detected in regions centred on the areas of ligand expression (Figure 3C). Furthermore, these regions are larger than the area of detectable *upd* expression, a finding that is consistent with Upd being a diffusible molecule able to trigger pathway activity non-autonomously [7], [8]. In *os^o^* mutant wing discs, *10xSTATGFP* activity is not detected in the proximal fold (arrow in Figure 3D). This result is consistent with the *upd in situ* data and also suggests that no other *upd*-like genes able to stimulate the JAK/STAT pathway are expressed in this domain in *os^o^* mutants.

**Figure 4:**
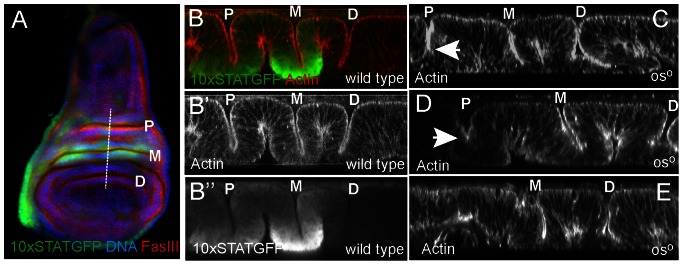
JAK/STAT signalling and fold formation. Proximal (P), medial (M) and distal (D) folds are labelled. (**A**) Wild type third instar wing imaginal discs showing the pattern of *10xSTATGFP* reporter activity (green), FasIII (red) and DNA (blue). The approximate position of the XZ sections shown in B–E is indicated by the dotted line. (**B**) An XZ section through the lateral wing folds of wild type third instar wing imaginal disc showing F-actin (red in B and white in B’) and the 10xSTATGFP reporter (green in B and white in B”). (**C–E**) XZ sections through third instar wing imaginal discs of males hemizygous for the *os^o^* allele stained to visualise F-actin (white), and hence disc structure. The P fold is reduced in depth in C and D (arrows) and is missing completely in E.

One of the more noticeable features of the third instar wing imaginal disc are three prominent lateral folds, which we term the proximal (P), medial (M) and distal (D) folds (Figure 3 and 4). These folds are present within the presumptive dorsal hinge between the wing pouch and the future notum, and run parallel to one another across both anterior and posterior compartments of the disc (Figure 3E). Expression domains of *upd* during late third instar development coincide with the M and P folds (Figure 3A), as does *10xSTATGFP* reporter expression (Figure 3C) while *upd* is also expressed in this general domain at earlier stages before folds are formed [24]. Consistent with the change in *upd* expression and JAK/STAT pathway activity in *os^o^* mutants described above, fate mapping experiments have previously identified the AS1 as being derived from a region of the disc located in the middle of the P fold (Figure 3E) [25]. This correlation is both consistent with our own findings and also supports the accuracy of the original fate mapping experiments.

Taken together, our results suggest a link between the loss of the AS1 and loss of an *upd* expression domain in *os^o^* mutants.

### Wing Disc Folds

The P, M and D folds present within the third instar wing imaginal disc can be readily visualised both in plan (XY; Figure 4A) and cross section (XZ; Figure 4B). Viewed in cross section (Figure 4B), folds can be seen to result from a concertina-type arrangement of the columnar epithelium in this area, a morphology that also results in two additional ‘opposite’ folds on the basal surface. Within this structure, *10xSTATGFP* reporter activity is highest in the cells making up the basal portion of each fold with overall levels of pathway activation higher in the M than the P fold (Wells *et al* submitted; Figure 4B”). Given the diffusible nature of the Upd ligand, this localised JAK/STAT pathway activation may be a result of ligand trapping within the fold. By contrast, and consistent with the lack of *upd* expression in this region, no reporter activity is detected in the D fold.

While all three folds are consistently of comparable depth in wild type discs (Figure 4B) the P fold in *os^o^* individuals is often disrupted (Figure 4C–E). Although somewhat variable, the P fold phenotype in this background can include a reduction in fold depth (Figure 4C), a reduced depth combined with an apical widening of the fold (Figure 4D) or even complete loss of the structure (Figure 4E). It seems likely that variations in P fold morphology and the variable loss of structures within the adult wing hinge derived from this region of the disc are related, with more severe fold phenotypes possibly resulting in more extensive loss of hinge structures.

### Modulation of STAT92E can Reproduce the Outstretched Phenotype

**Figure 5:**
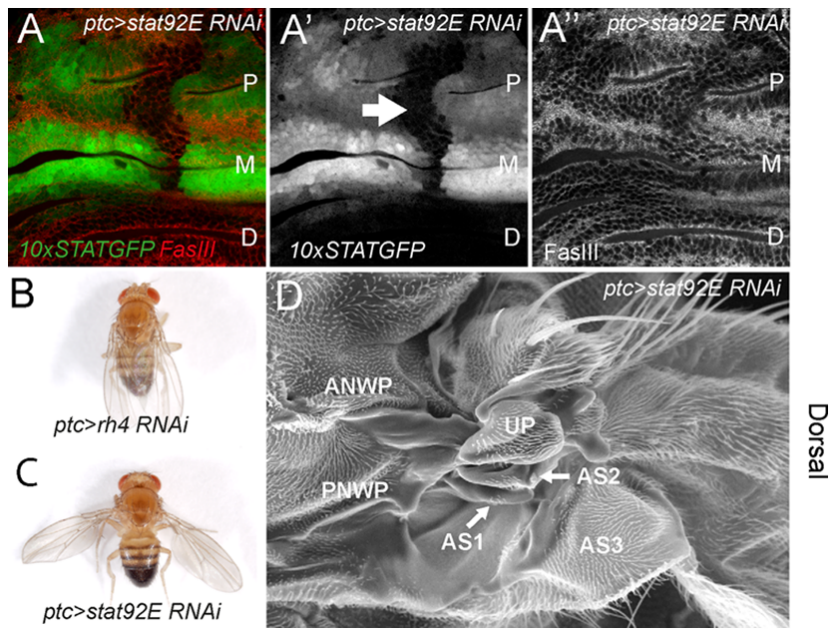
Modulation of JAK/STAT activity phenocopies *os* defects. (**A**) Dorsal hinge region of a third instar wing imaginal disc from a *10xSTATGFP, ptc>Gal4/UAS-stat92E RNAi* individual showing the extent of 10xSTATGFP reporter activity (green in A, white in A’) and FasciclinIII (red in A, white in A”) to outline cell shape. P, M and D folds are labeled and the domain in which reporter activity is ablated following knockdown of *stat92E* is shown (arrow in A’), (**B–C**) Adult male flies of the indicated genotypes showing resting wing posture. Knockdown of *stat92E* mRNA in the *ptc* domain produces a clear and highly penetrant wings held out defect. (**D**) SEM of the dorsal wing hinge of a *ptc>stat92E RNAi* male with a wings held out phenotype. Compared to a wild type hinge (Figure 2A) both the first and second auxiliary sclerites (AS1 and AS2) are reduced.

The link between the *os^o^* phenotype, *upd* expression and the morphology of the AS1 in the adult suggests that the *os* wing posture phenotype results from a defect in JAK/STAT signalling. In order to test this we used the *10xSTATGFP* pathway reporter recombined with a *patched (ptc)* transgene expressing Gal4 in a stripe of cells running through the middle of presumptive dorsal hinge region. Using this driver, we expressed an *in vivo* RNAi hairpin targeting the single *stat92E* transcript (Figure 5A) and find that activity of the *10xSTATGFP* pathway reporter is down regulated in a domain that intersects both the M and P folds (arrow in Figure 5A’). This demonstrates the extent of the *ptc* expression domain, the efficiency of knockdown and, as previously shown in Wells *et al* (submitted), also results in disruption of P fold morphology. Having established the extent of JAK/STAT pathway knockdown we allowed individuals to develop to adulthood. While a control RNAi targeting *Rhodopsin4 (Rh4),* a gene not expressed in the wing, shows no adult wing posture phenotype (Figure 5B), knockdown of *stat92E* results in a held out wing phenotype reminiscent of *os^1^* and *os^o^* (Figure 5C). When examined by SEM, *ptc>stat92E RNAi* flies show defects in the dorsal hinge with a reduction in both the AS1 and AS2 (Figure 5D). These defects are comparable to the original *os^1^* allele (Figure 2) and suggest that the wing posture phenotype in both genetic backgrounds are caused by the same fundamental mechanism.

In summary, we present the first detailed morphological and gene expression data of the classical group of *outstretched (os)* alleles – viable alleles of the *unpaired* locus. We go on to show that pathway activity in the proximal (P) lateral fold normally present in the third instar wing disc, is required for the normal development of this fold, and that a reduction of JAK/STAT pathway signalling in this fold is sufficient to phenocopy the *os* wing posture defect.

## Materials and Methods

### Drosophila Stocks and Genetics

*Drosophila* stocks were maintained on standard food at 25°C unless otherwise stated. Stocks used were *white^1118^ (w^1118^)* as a wild type control, *w,os^1^* and *f,os^o^,car* (gift of the Perrimon lab, Harvard Medical School) and *os^s^* (Bloomington Stock Centre – stock 79). *10xSTATGFP* reporter lines were a gift of Erika Bach (NYU Langone Medical Centre). Recombinants between *10xSTATGFP* [23] and *ptc>Gal4* [26] were generated using standard techniques. The *in vivo* RNAi constructs targeting *stat92E* mRNA (ID 106980) and *Rh4* mRNA (ID 101708) [27] were obtained from the Vienna *Drosophila* RNAi Centre.

### Scanning Electron Microscopy

Scanning electron micrographs were prepared from representative males flies of the indicated genotypes. In order to maintain normal live wing posture flies were rapidly killed in a petri dish on dry ice for 90 minutes, before being placed on 12 mm round Leit Adhesive Carbon Tabs (Agar Scientific). Flies were handled via their legs to ensure minimal disturbance of wing position. Samples were mounted onto 0.5 inch aluminium studs and gold coated using an Edwards S1501b sputter coater (Edwards, Crawley). Samples were examined in a Philips SEM XL-20 with an operating voltage of 20 kv. Pictures were taken at a working distance of between 9.8 and 10.2 mm with a magnification of 400x.

### Histology and Microscopy

Wing imaginal discs were dissected from wandering third instar larvae of the indicated genotypes. Whole mount *in situ* hybidisation to wing imaginal discs was undertaken as described [24] using antisense RNA probes generated from Upd cDNA plasmids (gift of Norbert Perrimon, Harvard Medical School). Immunostaining of imaginal discs was undertaken as described in [8]. Discs were counterstained to visualise DNA and filamentous-actin (F-actin) according to standard protocols using DAPI or Phaloidin-Alexa568 respectively. Antibodies used include mouse anti-Fasciclin III (1∶10), (Developmental Studies Hybridoma Bank). Secondary antibodies were from Jackson Labs.

Whole flies were photographed using a Nikon SMZ1500 stereo microscope and Nikon Elements extended depth of focus software package. Imaginal discs with *in situ* hybridisation patterns and sections through intact flies were imaged using differential interference contrast or bright field techniques on a Zeiss Axioskop microscope with Q-Imaging camera system. Zeiss LSM510 or Leica SP confocals were used to capture XY and XZ sections of imaginal discs containing fluorescent proteins and stains.

Longitudinal sections of adult flight muscles flies were generated from male *w^1118^* and *parkin^25^* mutants (used as controls [20]) and hemizygous *os^0^* and *os^1^* males. Individuals were anesthetised before partial dissection to remove legs, wings, proboscis and the posterior abdomen. Individuals were then fixed in 10% formaldehyde in PBS for 1 hr before dehydration through a series of ethanol dilutions (30%, 50%, 70%, 90% and 100% x 3) for 15 minutes at each step. Flies were then incubated over night in JB4 (Polysciences Inc) solution A combined with 18 mg/ml of catalyst (benzoyl peroxide) before being arranged into embedding moulds containing embedding solution mixture (JB4 solution A, 18 mg/ml of catalyst and 1% JB4 solution B). Block holders were added to exclude oxygen during the polymerisation process. Moulds were incubated at 4°C overnight before 3–5 µm sections were cut using a glass blade on a LKB microtome (Bromma, Sweden) and stained using 1% Tolundine blue/1% Na-Borax for 1 minute before being washed with water and dried. Coverslips were mounted using DPX before imaging.
